# Cost-effectiveness analysis of anidulafungin in the treatment of candidaemia

**DOI:** 10.1186/cc12025

**Published:** 2013-03-19

**Authors:** G Auzinger, G Playford, C Graham, H Narula, C Charbonneau, D Weinstein, M Kantecki, H Schlamm, M Ruhnke

**Affiliations:** 1 King's College Hospital, London, UK; 2 Princess Alexandra Hospital, Brisbane, Australia; 3 RTI Health Solutions, Durham, NC, USA; 4 Pfizer International Operations, Paris, France; 5 Pfizer Inc., New York, NY, USA; 6 Charité University Medicine, Berlin, Germany

## Introduction

Echinocandins are recommended first-line treatment for candidaemia [1]. A cost-effectiveness model developed from a UK perspective examined costs and outcomes of antifungal treatment for candidaemia and other forms of invasive candidiasis based on European clinical guidelines [1].

## Methods

Costs and treatment outcomes with the echinocandin anidulafungin were compared with caspofungin, micafungin, fluconazole, voriconazole and amphotericin B. The model included non-neutropenic patients aged ≥16 years with confirmed candidaemia/another form of invasive candidiasis receiving intravenous first-line treatment [2]. Patients were categorised as a clinical success or failure (patients with persistent/breakthrough infection); frequency data for each outcome were taken from a mixed-treatment comparison [3]. Successfully treated patients switched to oral therapy. Clinical failures switched to a different antifungal class. It was assumed that second-line treatment duration was equivalent to that of first-line treatment and only two lines of therapy were required to treat infection. Other inputs were all-cause 6-week mortality, cost of treatment-related adverse events (AEs) and other medical resource use costs. Life-years were calculated using a published model [4]. Antifungal agent-related AEs were taken from the product label/literature. Resource use was derived from the literature and discussion with clinical experts. Drug acquisition/ administration costs were taken from standard UK costing sources.

## Results

First-line anidulafungin for treatment of candidaemia was cost-effective per life-year gained versus fluconazole (incremental cost-effectiveness ratio £813). Anidulafungin was cost saving versus caspofungin and micafungin in terms of life-years gained due to lower ICU costs and a higher rate of survival combined with a higher probability of clinical success.

## Conclusion

Anidulafungin was cost-effective compared with fluconazole for treatment of candidaemia and was cost saving versus other echinocandins in the UK. European guidelines recommend echinocandins as first-line treatments for candidaemia [1]; this model indicates that anidulafungin marries clinical effectiveness and cost-effectiveness.
